# Effects of Expanding Infection Control Team Functions on Device-Associated HAIs: A Leadership-Oriented Intervention Study (2017–2024)

**DOI:** 10.3390/jcm15062168

**Published:** 2026-03-12

**Authors:** Marta Wałaszek, Piotr Serwacki, Wioletta Świątek-Kwapniewska, Róża Słowik, Piotr B. Heczko, Jadwiga Wójkowska-Mach

**Affiliations:** 1 Faculty of Medicine and Health Sciences, University of Tarnów, Mickiewicza 8, 33-100 Tarnów, Poland; walaszekm@lukasz.med.pl (M.W.); wkwapniewska@lukasz.med.pl (W.Ś.-K.); 2 St. Luke’s Provincial Hospital, 178A Lwowska Street, 33-100 Tarnów, Poland; pserwacki@lukasz.med.pl (P.S.); rslowik@lukasz.med.pl (R.S.); 3 Department of Microbiology, Faculty of Medicine, Jagiellonian University Medical College, Czysta 18, 31-121 Kraków, Poland

**Keywords:** healthcare-associated infections, device-associated infection, infection prevention interventions, leadership, communication

## Abstract

**Background/Objectives:** The effective prevention and control of healthcare-associated infections (HAIs) require the active engagement of clinical staff, which depends on strong relationships between the Infection Prevention and Control Team (IPCT) and frontline healthcare personnel. The role of the Infection Control Physician (ICP) as a clinical leader is essential for supporting evidence-based practice and fostering collaboration. This study aimed to demonstrate the impact of leadership-oriented interventions—particularly the introduction of ICP consultations in hospital wards—on HAI surveillance quality. **Methods:** A retrospective observational quasi-experimental study was conducted in a single hospital in southern Poland between 2017 and 2024, excluding 2020–2021 due to the COVID-19 pandemic. HAI surveillance followed the ECDC HAI-Net methodology. The study included all hospitalized patients in wards where invasive medical devices or invasive procedures were used. The intervention consisted of expanding the IPCT, increasing managerial support, extending infection control nurses’ competencies, and implementing routine ICP medical consultations. Changes in HAI incidence rates between the pre-intervention (pre-IP) and post-intervention (post-IP) periods were analyzed for catheter-associated urinary tract infections (CAUTI), ventilator-associated pneumonia (VAP), and central line-associated bloodstream infections (CLABSI), expressed per 1000 device-days. **Results:** The overall device utilization increased from 0.44 to 0.54 per 1000 patient-days in the post-IP period. The utilization of microbiological diagnostic tests more than doubled, with marked increases in blood cultures (6.4% vs. 15.5%) and urine cultures (7.7% vs. 11.0%). No IPCT consultations occurred in the pre-IP period, while 874 consultations were recorded in the post-IP period. Th incidence rates for CAUTI and VAP increased (1.4 to 3.1 and 11.7 to 24.6 per 1000 device-days, respectively). The CLABSI incidence showed no significant overall change. **Conclusions:** Structural and functional changes in the IPCT, combined with the introduction of ICP consultations, substantially enhanced the quality and completeness of HAI surveillance in the analyzed hospital. The findings highlight the importance of leadership-driven engagement in improving infection prevention and control systems.

## 1. Introduction

Healthcare-associated infections (HAIs) continue to pose a major threat to patient safety and healthcare quality worldwide. The effective prevention and control of HAIs require not only evidence-based protocols but also the active engagement of all healthcare personnel and strong interdisciplinary collaboration within hospital settings. The Infection Prevention and Control Team (IPCT) is central to these efforts, fostering a culture of safety, accountability, and continuous improvement across clinical departments. A positive safety culture has been consistently associated with improvements in infection prevention and control (IPC) practices and reductions in HAI rates [1]. According to Cen et al., IPC safety culture, understood as a multidimensional organizational climate, significantly affects healthcare workers’ adherence to IPC measures. Enhancing basic IPC competence, hospital management support, organizational learning, reporting practices, and targeted interventions for clinical instructors can improve compliance and help prevent HAIs [2].

The core responsibilities of the IPCT include designing and implementing comprehensive HAI surveillance systems, facilitating the ongoing education of healthcare staff, and ensuring transparent communication between clinical teams and infection control experts. Surveillance systems form the backbone of IPC efforts: by systematically collecting data on HAI incidence, microbiology, and antimicrobial resistance, IPCTs can perform epidemiological analyses that pinpoint infection trends and guide targeted interventions. Microbiological laboratories contribute critically to this process by identifying pathogens, detecting resistance patterns, and supporting outbreak investigations—enabling timely and precise infection control actions, including outbreak detection and response [3,4,5]. Interpreting surveillance data and calculating incidence rates are essential not only for local risk assessment and intervention but also for benchmarking hospital performance against regional, national, and international standards. Such benchmarking allows IPCTs to contextualize their outcomes, set realistic targets, and systematically improve IPC practices [6].

In the 2022–2023 ECDC point-prevalence survey across 93 Polish acute-care hospitals, the overall prevalence of healthcare-associated infections (HAIs) was 5.7% (1358/23,661), with the highest burden in ICUs (28.2%). Over half of HAIs had microbiological confirmation (55.1%, 804/1458), enabling organism-level characterization. The leading bacterial pathogens causing HAIs included *Enterobacterales* (notably, *Escherichia coli*, *Klebsiella* spp., and *Enterobacter* spp.) alongside *Staphylococcus aureus*, *Enterococcus* spp., *Pseudomonas aeruginosa*, and *Acinetobacter baumannii*, and *C. difficile* infections accounted for 8.2% of all HAIs. Third-generation cephalosporin resistance among *Enterobacterales* reached 41.4% overall (26.9% in *E. coli* and 59.1% in *Klebsiella* spp.). Carbapenem resistance was 9.4% in *Enterobacterales* overall (3.6% *E. coli* and 17.6% *Klebsiella* spp.) and was higher in non-fermenters (35.3% *P. aeruginosa* and 71.7% *A. baumannii*). Among the Gram-positives, 19% of S. aureus were MRSA and vancomycin resistance in *Enterococci* was 25.9% overall (45.1% in *E. faecium*), underscoring the clinical impact of bacterial HAIs in Poland [7].

Unfortunately, these data come from a point prevalence study, as Polish hospitals rarely participate in continuous multicenter surveillance. According to ECDC data, the participation of Polish hospitals in pan-European surveillance networks is markedly lower than the European average: 5.4% vs. 57.4% overall, respectively; 5.9% vs. 23.3% for surgical site infection surveillance, respectively; 4.9% vs. 28.6% for ICU HAI surveillance, respectively; and 4.4% vs. 46.2% for *C. difficile* infection surveillance, respectively [7].

Infection surveillance in Poland is significantly influenced by historical context. In 1989, a political transformation led to the adoption of a more open approach to detecting HAIs—something that was previously hindered by structural and political barriers. Detection was possible but surveillance involving analysis and feedback was not. The Polish surveillance system was modeled after systems that had been developed in the USA and the UK since the 1990s. Today, each hospital in Poland has an IPCT, typically composed of an infection control nurse (ICN) and an infection prevention physician (ICP).

On the other hand, equally important are the organizational structure and organization of HAI control and support from management at the hospital level [8]. However, crucial to maintaining the lasting effects of HAI surveillance are the identification and maintenance of formal and informal leadership and change leaders [9]. A qualitative study conducted in European hospitals on the implementation of HAI prevention practices confirmed that successful implementation required having sufficient human and material resources and dedicated change agents who helped make the intervention an institutional priority [10]. The personal commitment of influential individuals and boundary spanners helped overcome resource restrictions and intra-institutional segregation [10].

Knobloch et al. emphasized the essential role of implementing evidence-based infection prevention practices, which require not only technical competence but also effective leadership and strong relational engagement with both frontline healthcare workers and hospital management [11]. Saint et al. demonstrated that hospital epidemiologists and infection preventionists frequently occupy more influential leadership positions in patient safety initiatives than senior executives themselves, underscoring the uniquely strategic role of infection prevention professionals in shaping safety culture [12]. Furthermore, according to Knobloch et al., successful leaders share several defining characteristics: (1) they cultivate a culture of clinical excellence and communicate it clearly and consistently; (2) they address barriers directly, including resistance among staff or workflow problems that hinder HAI prevention; (3) they inspire and motivate their teams; and (4) they think strategically at the organizational level while acting effectively at the local, unit-based level [11].

In relation to the concept of cultivating a culture of clinical excellence, Flodgren et al. highlighted the importance of local opinion leaders (OLs)—trusted and credible individuals who promote and disseminate best evidence, often through informal one-to-one interactions or targeted educational outreach activities [13]. These OLs serve as critical intermediaries between evidence and practice, helping to embed standards of excellence into daily clinical routines.

Moreover, effective HAI prevention requires active collaboration between the ICP as a clinical leader and hospital executives. As demonstrated by the findings of McAlearney et al., such collaboration must be operationalized through three key leadership practices: (1) engagement and visible commitment from executive leadership; (2) open and structured information sharing across organizational levels; and (3) coaching and mentorship provided by managers to support frontline teams in sustaining prevention efforts [14]. Together, these elements form the leadership framework necessary for driving meaningful and sustained improvements in HAI prevention.

In our study, the leadership qualities of a leader were demonstrated by an infection control physician (ICP) who built interpersonal relationships. However, the analysis of IPCT social networks allowed us to identify IPCT connections with high-ranked hospital employees and with influential people from outside the hospital associated with the world of science who helped promote solutions and make decisions appropriate to the development of an effective HAI control system. In Poland, building relationships based on safety and the analysis of social networks is important due to cultural features such as high-power distance and high-uncertainty avoidance, which can influence the behavior of individuals. The aim of this study was, therefore, to assess the impact of the positive bias of leadership in nosocomial infection surveillance, reflected in changes in the detection and control of device-associated infections, resulting from the daily support provided to medical staff by the IPCT and consultations with the ICP during the period 2017–2024.

## 2. Materials and Methods

### 2.1. Setting

A single-center retrospective observational before–after quasi-experimental study was performed in the 650-bed St. Luke’s Provincial Hospital in southern Poland during the period 2017–2024. The years 2017–2019 were considered the pre-intervention period (pre-IP) and the years 2022–2024 were the post-intervention period (post-IP). During 2020–2021, due to the COVID-19 pandemic, the hospital was partially redesignated as a “COVID-only hospital” as many of the hospitals in Poland in that time period were also connected with a burden in the hospital infection, prevention, and control performance. Due to the above, the years 2020–2021 have been excluded from the analysis.

### 2.2. Study Design

Among all 18 hospital wards, 5 were included in the study. The inclusion criteria were having intensive care beds in the ward structure and patient treatment involving the use of medical devices and invasive procedures, as set out below:

- bladder catheterization
- and/or central venous catheterization
- and/or invasive mechanical ventilation

**Figure 1:**
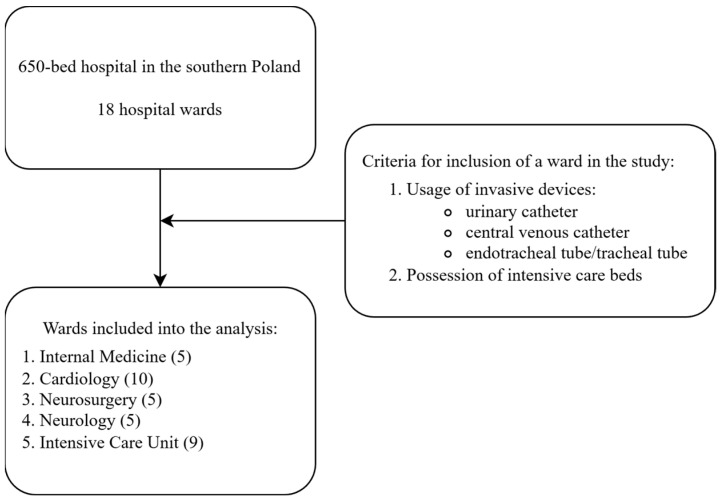
Criteria for the inclusion of hospital wards in the study according to use of the selected device and invasive procedures (in brackets, number of intensive care beds).

Data were collected from the following five wards: ICU, internal medicine, cardiology, neurosurgery, and neurology (Figure 1). Patients who underwent short-term procedures, such as urinary catheterization only for the duration of surgery, when the catheter was removed directly after the procedure, were excluded.

### 2.3. Infection Prevention and Control Team

In the studied hospital, the Infection Prevention and Control Team (IPCT) had been operating since 2001. Initially, the team consisted of minimal personnel: one infection control nurse (ICN), one infection control physician (ICP), and one microbiologist. In 2009, three trained ICNs were employed. The IPCT used an active-passive healthcare-associated infection (HAI) surveillance system based on microbiological test results. However, the hospital reported a significantly lower incidence of HAIs than expected for a population with comparable exposure risks; for example, in the internal medicine ward, where urinary catheters were routinely used, no cases of catheter-associated urinary tract infections (CAUTIs) were recorded. Therefore, the IPCT stated that this was related to insufficient detection of CA-UTIs.

In 2021, an ICP infectious disease doctor joined the IPCT and completed postgraduate studies in antibiotic stewardship. At the beginning of 2022, the IPCT welcomed a new member, a physician undergoing specialization in anaesthesiology and intensive care, who had also completed postgraduate education in infection prevention and control. Their combined clinical and epidemiological background significantly enriched the team’s competencies.

Alongside these personnel changes, modifications were introduced to the HAI surveillance system, including the implementation of a rational antibiotic policy and the provision of consultations by ICPs regarding the diagnosis and treatment of patients with infections.

**Figure 2:**
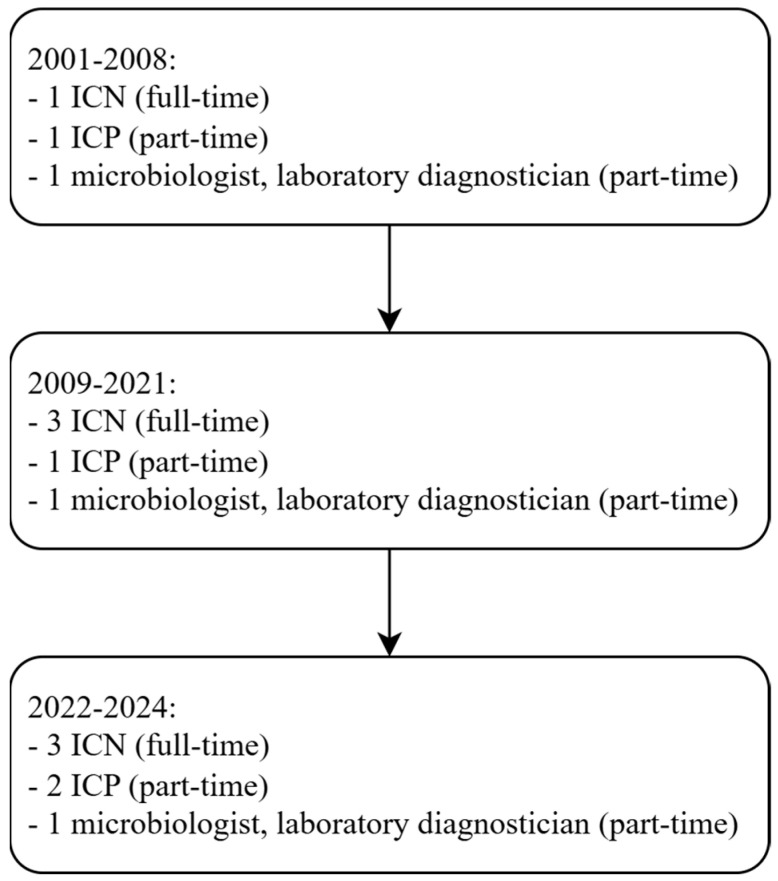
Changes in the composition of the Infection Prevention and Control Team (IPCT) for the years 2001–2024.

Starting in 2022, under the revised surveillance system, the authority to diagnose HAIs was extended to ICNs, ICPs, and all physicians across departments. During this time, the IPCT received support from hospital management, including financial support for the ICP and microbiologist. Although both were hospital employees, they were not full-time members of the IPCT (Figure 2).

In 2022, for the first time, medical consultations for patients with HAIs were formally introduced and provided by the ICPs. To assess the impact of these changes, HAI incidence rates associated with medical devices (urinary catheters, central venous catheters (CVCs), and endotracheal/tracheal tubes) were compared between the pre-IP (2017–2019) and post-IP (2022–2024). The results were expressed as incidence rates per 1000 patient-days for the following infections: catheter-associated urinary tract infections (CAUTIs), ventilator-associated pneumonia (VAP), and central line-associated bloodstream infections (CLABSIs).

The recognition and qualification of an HAI was realized according to the methodology recommended by ECDC HAI-Net [15]. To gain insight into the HAIs that occurred in connection with the use of invasive devices, the ECDC protocol was used [16].

The invasive device (CVCs, MVs, and urinary catheters) utilization ratio was calculated as the device days\patient days [17].

The intervention used included:

1. IPCT management:
  a. Inclusion in the IPCT of two physicians who were hospital employees—one infectious disease specialist and one in the course of anesthesiology and intensive care
  b. Completion of postgraduate studies by ICPs in the field of key work IPCT competencies
  c. Inclusion of a microbiologist and diagnostician in the IPCT (in the course of specialization in microbiology)
  d. The award of additional compensation for work in an IPCT to the ICPs and the microbiologist
2. Formal and informal support from hospital management for the work of the IPCT
3. Granting HAI recognition authority to ICNs and the hospital department’s physicians
4. Introduction of ICP medical consultations in cases of infection or suspected infection in the range of:
  a. Diagnostics, including microbiological diagnostics
  b. Antibiotic treatment (empirical and targeted therapy)
5. Systematic building of a network of horizontal relationships with physicians in hospital wards by ICPs

To evaluate the impact of the implemented interventions in HAI surveillance, several indicators were analyzed, including the rate of microbiological test utilization, which reflected diagnostic activity and the clinical suspicion of infection, as well as the incidence rates of detected infections. Particular attention was given to device-associated infections (DAI), specifically, ventilator-associated pneumonia (VAP), central line-associated bloodstream infections (CLABSI), and catheter-associated urinary tract infections (CAUTIs). Medical device utilization, the measurement of how often medical devices like catheters, ventilators, and central lines were used compared to patient availability, were calculated as the device-days/patient-days. Microbiological test utilization rate was defined as the number of selected microbiological diagnostic tests of clinical material (without screening test) performed per 1000 patient-days in a healthcare facility over a specified period.

For the statistical analysis of the collected material, IBM SPSS (SPSS-Statistical Package for the Social Sciences, STATISTICS 24, Armonk, NY, USA) and Microsoft Excel (Microsoft Office 2016 Redmond, WA, USA) were used. The following were calculated in the statistical analysis: incidence rate ratio (IRR), standardized infection ratios (SIR), incidence rate per 1000 pds per catheter-associated device-use (CA), mechanical ventilation (MV), central venous catheter (CVC), frequency (n), percent (%), significance level, and 95% confidence interval (95% CI). To analyze the relationship between the number of HAIs and the number of cultures and consultations, a negative binomial regression model was applied due to the presence of overdispersion in the count data. The presence of overdispersion was confirmed by comparing the Poisson model with the negative binomial model and by estimating the dispersion parameter (α > 0). A multivariable negative binomial regression model was constructed, including the HAI (categorical variable), number of cultures, and number of consultations as independent variables. The model intercept described the baseline level of HAIs when all predictors were 0. The results are presented as regression coefficients (B) and incidence rate ratios (IRR, Exp(B)), with 95% confidence intervals (95% CI) and *p*-values. For variables such as the number of cultures and consultations, the results were interpreted as the percentage change in the event counts according to the formula (IRR, Exp(B) − 1) × 100%. A *p*-value of < 0.05 was considered statistically significant.

## 3. Results

### 3.1. Patient Admissions and Device Utilization

Across the five hospital departments, a total of 29,059 admissions were recorded during the pre-IP and 23,772 admissions post-IP. The distribution of admissions across departments remained broadly similar, although the proportion of admissions to the Intensive Care Unit (ICU) increased from 3.7% to 5.4%. Device utilization increased across all device types. The number of UC-pds rose from 50,189 to 52,997; MV-pds increased from 5731 to 6755; and CL-pds increased from 13,499 to 15,046. The overall device utilization rate increased from 0.44 to 0.54 per 1000 pds. The utilization of microbiological diagnostics increased substantially. Overall microbiological testing rose from 13.9 to 29.9 per 100 admissions from the pre-IP to the post-IP. The largest proportional increase was observed in blood cultures, which nearly doubled (from 1860 to 3680 sets). The proportion of negative blood cultures decreased slightly from 96.89% in the pre-IP to 88.87% in the post-IP (Table 1).

### 3.2. Incidence Rates of Device-Associated HAIs

Across all departments, CA-UTI incidence increased from 1.4 to 3.1 per 1000 UC-pds, corresponding to an overall IRR of 2.21 (95% CI: 2.44–4.38; *p* < 0.001); in particular, rises were observed in cardiology (IRR 11.0 (*p* < 0.001)), neurology (IRR 2.1 (*p* < 0.001)), and ICU (IRR 2.21 (*p* < 0.01)). VAP incidence increased from 11.7 to 24.6 per 1000 MV-pds, with an IRR of 2.1 (95% CI: 2.29–4.04; *p* < 0.001). The highest relative increases occurred in cardiology (IRR 15.1 (*p* < 0.001)), neurology (IRR 8.8 (*p* < 0.001)), and neurosurgery (IRR 2.1 (*p* < 0.001)). Overall CLABSI incidence remained stable (4.9 vs. 4.3 per 1000 CL-days; IRR 0.9; *p* = 0.36) except in cardiology (IRR 13.1; *p* < 0.01), where the incidence rate increased, and ICU, where it decreased (IRR 0.6; *p* < 0.05, Table 2).

The overall standardized infection ratios (SIR) for CA-UTI increased to 2.6, with the highest rates in cardiology (11.2). VAP SIRs also increased, with an overall SIR of 2.1. Extremely elevated SIRs occurred in internal diseases (15.1) and neurosurgery (8.8). CLABSI SIRs displayed large departmental variation, with 13.1 in internal diseases, 4.4 in neurosurgery, and below 1 in both ICU and neurology (0.6) (Table 3).

### 3.3. Negative Binomial Regression Analysis

The negative binomial regression assessed the association between HAI counts and two predictors: microbiological test utilization and infection control physician (ICP) consultations. Across all departments, microbiological test volume showed no significant association with HAI incidence, and the total model IRR was 1.00 (95% CI: 1.000–1.000; *p* = 0.536). Department-level coefficients were consistently close to 1.00, with nonsignificant *p*-values. However, ICP consultations showed significant positive associations with HAI counts in two departments: neurosurgery (IRR 1.010 (95% CI: 1.005–1.016; *p* < 0.001)) and neurology (IRR 1.015 (95% CI: 1.008–1.022; *p* < 0.001)), without associations in other departments. In the overall model, consultations were not significantly associated with HAI frequency (IRR 1.001; *p* = 0.494, Table 4).

## 4. Discussion

The present study demonstrated substantial increases in device-associated infection rates and standardized infection ratios across several hospital departments following the intervention period. Although microbiological test utilization and infection control consultations expanded considerably, the negative binomial regression analyses revealed no meaningful association between test volume and HAI burden and only limited, department-specific correlations with consultation frequency. These findings indicate that increased diagnostic and consultative activities alone were insufficient to reduce HAI incidence—and therefore, the sensitivity of the surveillance expressed by the increase in detected HAI cases—and highlight the need to reassess the effectiveness, implementation fidelity, and contextual fit of infection prevention strategies during the study period.

The observed post-intervention period saw increases in the numbers of microbiological tests and IPCT consultations in our hospital, which likely reflected the heightened vigilance and closer bedside engagement, both of which are prerequisites for effective HAI prevention and timely response. At the same time, the concomitant increases in CAUTI and VAP applications required targeted action. For CAUTI, very low benchmark rates have been documented in German ICUs participating in the national KISS surveillance [18], underscoring what is achievable under mature surveillance and prevention programs. In contrast, large international, multicenter studies have focused predominantly on ICU patient reported pooled CAUTI burdens that were closer to our experience (INICC: ~2.8 per 1000 UC-pds), highlighting the wide international spread attributable to differences in device exposure, diagnostic practices, and resource constraints [19]. A similar picture has emerged for VAP: our post-intervention rate sat above what would be expected based on European multicenter estimates (EU-VAP: 18.3 per 1000 MV-pds) [20], reinforcing the need to intensify ventilator bundle adherence, device-necessity reviews, and early weaning strategies in our setting. Collectively, these comparisons suggest that sustaining high diagnostic vigilance should be coupled with device-use optimization and diagnostic stewardship so that rising detection does not translate into preventable device-associated harm. Unfortunately, the CLABSI incidence rate, which remained unchanged, was also alarmingly high, approximately twice as high as the data from the German KISS system [21].

This suggests that while surveillance and communication improved, sustaining optimal device usage practices remained critical. Previous studies have highlighted that increases in test utilization often accompany more proactive, but sometimes disproportional, device use—underscoring the importance of coupling surveillance with targeted reduction efforts and stewardship initiatives [22]. Device-associated infections, particularly CAUTI, VAP, and CLABSI, continue to contribute substantially to morbidity in hospitalized patients despite concerted prevention efforts. Bundled strategies—such as those recommended by SHEA/IDSA/APIC [23]—have demonstrated efficacy in reducing CLABSI and CAUTI by more than 50% in some settings in the USA [22]. The lack of significant change in total CLABSI rates in this study may indicate an uneven adoption of the bundled elements across the departments. Department-specific reductions in CLABSI (e.g., neurosurgery) might reflect variations in protocol fidelity, reinforcing the need for standardized implementation and compliance monitoring across all units.

The integration of positive deviance (PD) principles strengthened by the leadership of the IPCT offers a promising strategy to reinforce IPC behaviors within complex care environments. Systematic reviews have shown consistent improvements in hand hygiene compliance and significant reductions in MRSA and, overall, HAI rates following PD implementation. Interventional studies have translated these PD frameworks into practice, demonstrating improved safety culture, the sustainable adoption of IPC norms, and tangible decreases in infection rates. Applying PD in our context—by identifying and scaling context-specific best practices for catheter maintenance or ventilator care—could strengthen interprofessional collaboration and enhance care processes that impact DAI incidence [24,25].

Benchmarking against internal and external standards remains essential for continuous IPC improvement. Global IPC networks and WHO guidelines emphasize surveillance and national benchmarking through initiatives like *IPC Assessment Framework* IPCAF—a structured, self-administered, validated tool that assesses a detailed list of 81 indicators related to the IPC core components—which enable hospitals to gauge performance and target underperforming areas. By analyzing increases in device utilization and correlating them with infection trends, the IPCT can identify priority areas for behavior change and resource allocation [5].

The strengths of our study include the comprehensive evaluation of device utilization, microbiology testing, IPC engagement, and DAI incidence across multiple departments, offering a multifaceted view of IPC impact. However, the limitations include its observational design, potential confounding factors such as staffing changes and patient case-mix, and the lack of qualitative assessment of IPC practices and organizational culture. These aspects are especially relevant given the evidence linking IPC staffing ratios with HAI rates.

On the other hand, it is worth emphasizing another positive effect of the ICP’s consultations introduction not shown in this study: the more rational use of antimicrobials in the post-IP compared to the pre-IP and years earlier. Namely, between 2012 and 2018, the surveillance of *C. difficile* infections (CDIs) in adult surgical wards in the studied hospital demonstrated a positive correlation between the use of fluoroquinolones and the incidence of CDI [26]. In the following years, the ICP’s consultations led to a significant reduction in the consumption of fluoroquinolones in these wards [27].

## 5. Limitations

This study had several important limitations. First, its retrospective design conducted in a single hospital limits the generalizability of the findings and introduces the possibility of unmeasured confounding, as no patient-level clinical factors were available to adjust for differences between the pre- and post-intervention periods. Second, the absence of data on pathogen profiles and antimicrobial resistance patterns restricts the interpretation of microbiological changes over time. Third, the before–after, quasi-experimental design did not include adjustments for patient case-mix or illness severity, and no standardized severity scores (such as APACHE II for ICU patients) were collected. In addition, no multivariate models were performed to adjust for device utilization or other structural confounders. These limitations should be considered when interpreting the observed changes in diagnostic activity and HAI indicators.

## 6. Conclusions

Introduced changes in the IPCT structure and functionality and the newly introduced ICP’s consultations markedly improved HAI surveillance in the analyzed hospital. Despite this positive phenomenon, sustained preventive measures should be taken in all the departments studied due to the continuing large differences in local incidence rates compared to those reported in European countries. To address rising CAUTI and VAP rates, hospitals should consider combining surveillance with proactive measures, such as daily device necessity reviews and graduated removal protocols within a bundled strategy. Embedding PD techniques—such as the frontline-led identification of barrier solutions to device care—can foster sustainable practice changes. In addition, structured benchmarking and regular feedback loops, supported by adequate IPC staffing, are crucial for sustaining improvements in patient safety culture and IPC outcomes. Leadership plays an important role in infection prevention and control. The behaviors of effective leaders can be emulated by others who strive to control infections.

## Figures and Tables

**Table 1:** Characteristics of the studied hospital departments before and after the intervention.

Studied Subpopulations	Intervention Comparison Periods	
	Pre-IP	Post-IP
**Patients’ admissions [N (%)]**		
Internal diseases	5947 (20.5)	4846 (20.4)
Cardiology	8018 (27.6)	7334 (30.9)
Neurosurgery	6147 (21.2)	5042 (21.2)
Neurology	7863 (27.1)	5271 (22.2)
ICU	1084 (3.7)	1279 (5.4)
TOTAL	29,059 (100.0)	23,772 (100.0)
Medical device utilization [N (device use rate *)]		
pds with urinary catheter	50,189 (0.32)	52,997 (0.38)
pds with mechanical ventilation	5731 (0.04)	6755 (0.05)
pds with central line	13,499 (0.09)	15,046 (0.11)
TOTAL	69,419 (0.44)	74,798 (0.54)
Microbiology test [N (test utilization rate **)]		
Urine cultures	1663 (7.7)	2619 (11.0)
BAL/tracheal aspirate	519 (1.8)	814 (3.4)
Blood cultures (sets)	1860 (6.4)	3680 (15.5)
TOTAL	4042 (13.9)	7113 (29.9)

* Device use rate: the number of device days (i.e., the total number of days a specific device was in use across all patients) divided by the total number of patient days, per 1000 patient days (pds); ** test utilization rate: microbiological test utilization per 100 admissions. Abbreviations: intensive care unit (ICU); preintervention period (pre-IP); postintervention period (post-IP).

**Table 2:** Incidence rate of the different types of device-associated infections across the studied hospital departments before and after the intervention.

Ward	CA-UTI Incidence Rate *				VAP Incidence Rate *				CLABSI Incidence Rate *			
	Pre-IP	Post-IP	IRR	OR (95% CI); *p*-Value	Pre-IP	Post-IP	IRR	OR (95% CI); *p*-Value	Pre-IP	Post-IP	IRR	OR (95% CI); *p*-Value
Internal diseases	1.0	3.9	3.9	(1.82–5.77); <0.001	7.6	34.9	4.6	(0.89–61.25); N/A	6.2	7.5	1.2	(0.58–4.09); 0.47
Cardiology	0.2	2.2	11.0	(3.71–20.43); <0.001	3.0	45.2	15.1	(2.17–124.42); <0.001	0.8	10.5	13.1	(1.76–115.02); <0.01
Neurosurgery	1.0	1.8	1.8	(1.06–4.64); 0.029	25.4	54.1	2.1	(1.63–5.91); <0.001	3.1	0.5	0.2	(0.05–1.13); 0.077
Neurology	1.4	2.9	2.1	(1.78–5.66); <0.001	5.6	49.1	8.8	(5.75–100.22); <0.001	1.8	8.0	4.4	(1.36–33.25); <0.01
ICU	2.4	5.3	2.21	(1.21–3.77); <0.01	11.4	16.0	1.4	(0.96–1.98); 0.081	6.9	4.1	0.6	(0.32–0.94); <0.05
TOTAL	1.4	3.1	2.21	(2.44–4.38); <0.001	11.7	24.6	2.1	(2.29–4.04); <0.001	4.9	4.3	0.9	(0.84–1.68); 0.36

* Incidence rate per 1000 pds per device use; abbreviations: intensive care unit (ICU); incidence rate ratio (IRR), pre-intervention period (pre-IP), post-intervention period (post-IP), catheter-associated urinary tract infections (CAUTIs), 95% confidence interval (95% CI), odds ratio (OR), ventilator-associated pneumonia (VAPs), central line-associated bloodstream infections (CLABSIs).

**Table 3:** Standardized risk index (SIR) of CA-UTIs, VAPs, and CLABSIs for 2017–2024, excluding the years 2020–2021, by selected hospital wards.

**CA-UTI—Standardized Risk Index**					
**Ward**	**Pre-IP** **incidence rate**	**Post-IP**			
		**Incidence rate per 1000 CApds**	**Observed cases [N]**	**Expected cases [N]**	**SIR**
Internal diseases	1.0	3.9	42	10.7	3.9
Cardiology	0.2	2.2	25	2.2	11.2
Neurosurgery	1.0	1.8	20	10.9	1.8
Neurology	1.4	2.9	36	17.3	2.1
ICU	2.4	5.3	42	19.0	2.2
TOTAL	1.2	3.1	165	63.6	2.6
**VAP—Standardized Risk Index**					
**Ward**	**Pre-IP** **incidence rate**	**Post-IP**			
		**Incidence rate per 1000 MVpds**	**Observed cases [N]**	**Expected cases [N]**	**SIR**
Internal diseases	3	45.2	15	1.0	15.1
Cardiology	25.4	54.1	33	15.5	2.1
Neurosurgery	5.6	49.2	32	3.6	8.8
Neurology	11.4	16.0	80	56.9	1.4
ICU	11.7	24.6	166	79.0	2.1
**CLABSI—Standardized Risk Index**					
**Ward**	**Pre-IP** **incidence rate**	**Post-IP**			
		**Incidence rate per 1000 CLpds**	**Observed cases [N]**	**Expected cases [N]**	**SIR**
Internal diseases	0.8	10.5	13	1.0	13.1
Cardiology	3.1	0.50	2	12.3	0.2
Neurosurgery	1.8	8.0	9	2.0	4.4
Neurology	6.9	4.1	30	50.9	0.6
ICU	4.9	4.3	64	73.7	0.9

**Table 4:** Negative binomial regression (NBR) results for healthcare-associated infection counts and predictors: microbiological testing and ICP consultations.

Ward	Microbiological Tests					ICP Consultations				
	B (SE)	IRR Exp (B) *	95% CI	%	*p*-Value	B (SE)	IRR Exp (B) *	95% CI	%	*p*-Value
Internal diseases	0 (0.001)	1	0.998–1.001	0.0	0.545	0.061 (0.040)	1.063	0.982–1.150	6.3	0.131
Cardiology	0 (0.001)	1	0.998–1.002	0.0	0.849	0.023 (0.023)	1.023	0.997–1.049	2.3	0.08
Neurosurgery	0.001 (0.001)	1.001	0.999–1.003	0.1	0.321	0.01 (0.010)	1.010	1.005–1.016	1.0	<0.001
Neurology	−0.001 (0.001)	0.999	0.998–1.000	−0.1	0.151	0.015 (0.003)	1.015	1.008–1.022	1.5	<0.001
ICU	0.001 (0.004)	1.001	1.000–1.001	0.1	0.217	0.002 (0.002)	1.002	0.994–1.010	0.2	0.604
TOTAL	0 (0.002)	1	1.000–1.000	0.0	0.536	0.001 (0.002)	1.001	0.998–1.004	0.1	0.494

* RR Exp(B): Calculated as the percentage change in the number of events using the formula ((IRR, Exp(B) − 1)) × 100. Abbreviations: healthcare-associated infection (HAI), infection control physician (ICP), regression coefficient (B), standard error (SE), 95% confidence interval (95% CI), Exp (B): incidence rate ratio.

## Data Availability

The data presented in this study are available on reasonable request from the first author. The data are not publicly accessible due to institutional privacy and data protection regulations.

## References

[1] Braun B.I., Chitavi S.O., Suzuki H., Soyemi C.A., Puig-Asensio M. (2020). Culture of Safety: Impact on Improvement in Infection Prevention Process and Outcomes. Curr. Infect. Dis. Rep..

[2] Cen Y., Lao C., Li Z., Zhao H., Wang T., Fan C., Liu B., Zhao Z., Zou Y., Lin G. (2025). Association between infection prevention and control safety culture and healthcare workers’ compliance with infection control measures: A cross-sectional study. Front. Public Health.

[3] Behnke M., Diaz L.P., Piening B., Pilarski G., Gastmeier P., Schröder C. (2015). Implementation of an electronic hospital outbreak detection system. Antimicrob. Resist. Infect. Control.

[4] Alshagrawi S., Alhodaithy N. (2024). Risk factors of healthcare-associated infection among healthcare workers in intensive care units: A multicenter cross-sectional study. PLoS ONE.

[5] World Health Organization (2024). Surveillance Of Health Care-Associated Infections at National and Facility Levels. https://www.who.int/publications/i/item/9789240101456.

[6] Zingg W., Holmes A., Dettenkofer M., Goetting T., Secci F., Clack L., Allegranzi B., Magiorakos A.-P., Pittet D. (2015). Hospital organisation, management, and structure for prevention of health-care-associated infection: A systematic review and expert consensus. Lancet Infect. Dis..

[7] European Centre for Disease Prevention and Control (2024). Point Prevalence Survey of Healthcare-Associated Infections and Antimicrobial Use in European Acute Care Hospitals.

[8] Hansen S., Zingg W., Ahmad R., Kyratsis Y., Behnke M., Schwab F., Pittet D., Gastmeier P., Sax H., Grundmann H. (2015). Organization of infection control in European hospitals. J. Hosp. Infect..

[9] Castro-Sánchez E., Holmes A. (2015). Impact of organizations on healthcare-associated infections. J. Hosp. Infect..

[10] Clack L., Zingg W., Saint S., Casillas A., Touveneau S., Jantarada F.d.L., Willi U., van der Kooi T., Damschroder L.J., Forman J.H. (2018). Implementing infection prevention practices across European hospitals: An in-depth qualitative assessment. BMJ Qual. Saf..

[11] Knobloch M.J., Thomas K.V., Musuuza J., Safdar N. (2019). Exploring leadership within a systems approach to reduce health care–associated infections: A scoping review of one work system model. Am. J. Infect. Control.

[12] Saint S., Kowalski C.P., Banaszak-Holl J., Forman J., Damschroder L., Krein S.L. (2010). The Importance of Leadership in Preventing Healthcare-Associated Infection: Results of a Multisite Qualitative Study. Infect. Control Hosp. Epidemiol..

[13] Flodgren G., O’Brien M.A., Parmelli E., Grimshaw J.M. (2019). Local opinion leaders: Effects on professional practice and healthcare outcomes. Cochrane Database Syst. Rev..

[14] McAlearney A.S., Gaughan A.A., DePuccio M.J., MacEwan S.R., Hebert C., Walker D.M. (2021). Management practices for leaders to promote infection prevention: Lessons from a qualitative study. Am. J. Infect. Control.

[15] European Centre for Disease Prevention and Control (2012). Point Prevalence Survey of Healthcare-Associated Infections and Antimicrobial Use in European Acute Care Hospitals—Protocol Version 4.3. https://www.ecdc.europa.eu/en/publications-data/point-prevalence-survey-healthcare-associated-infections-and-antimicrobial-use.

[16] European Centre for Disease Prevention and Control (2015). European Surveillance of Healthcare-Associated Infections in Intensive Care Units—HAI-Net ICU Protocol.

[17] Centers for Disease Control and Prevention, National Healthcare Safety Network (NHSN) (2024). The NHSN Standardized Utilization Ratio (SUR).

[18] Gastmeier P., Behnke M., Schwab F., Geffers C. (2011). Benchmarking of urinary tract infection rates: Experiences from the intensive care unit component of the German national nosocomial infections surveillance system. J. Hosp. Infect..

[19] Rosenthal V.D., Yin R., Brown E.C., Lee B.H., Rodrigues C., Myatra S.N., Kharbanda M., Rajhans P., Mehta Y., Todi S.K. (2024). Incidence and risk factors for catheter-associated urinary tract infection in 623 intensive care units throughout 37 Asian, African, Eastern European, Latin American, and Middle Eastern nations: A multinational prospective research of INICC. Infect. Control Hosp. Epidemiol..

[20] Koulenti D., Tsigou E., Rello J. (2017). Nosocomial pneumonia in 27 ICUs in Europe: Perspectives from the EU-VAP/CAP study. Eur. J. Clin. Microbiol. Infect. Dis..

[21] Geffers C., Schwab F., Behnke M., Gastmeier P. (2022). No increase of device associated infections in German intensive care units during the start of the COVID-19 pandemic in 2020. Antimicrob. Resist. Infect. Control.

[22] Saint S., Greene M.T., E Fowler K., Ratz D., Patel P.K., Meddings J., Krein S.L. (2019). What US hospitals are currently doing to prevent common device-associated infections: Results from a national survey. BMJ Qual. Saf..

[23] Yokoe D.S., Advani S.D., Anderson D.J., Babcock H.M., Bell M., Berenholtz S.M., Bryant K.A., Buetti N., Calderwood M.S., Calfee D.P. (2023). Executive Summary: A Compendium of Strategies to Prevent Healthcare-Associated Infections in Acute-Care Hospitals: 2022 Updates. Infect. Control Hosp. Epidemiol..

[24] Alzunitan M.A., Edmond M.B., Alsuhaibani M.A., Samuelson R.J., Schweizer M.L., Marra A.R. (2022). Positive deviance in infection prevention and control: A systematic literature review. Infect Control Hosp Epidemiol..

[25] Cohen R., Gesser-Edelsburg A., Singhal A., Benenson S., Moses A.E. (2022). Translating a theory-based positive deviance approach into an applied tool: Mitigating barriers among health professionals (HPs) regarding infection prevention and control (IPC) guidelines. PLoS ONE.

[26] Jachowicz E., Wałaszek M., Sulimka G., Maciejczak A., Zieńczuk W., Kołodziej D., Karaś J., Pobiega M., Wójkowska-Mach J. (2020). Long-Term Antibiotic Prophylaxis in Urology and High Incidence of Clostridioides difficile Infections in Surgical Adult Patients. Microorganisms.

[27] Serwacki P., Hareza D., Gajda M., Świątek-Kwapniewska W., Adamowska M., Serwacka K., Zawada G., Wałaszek M., Wójkowska-Mach J. (2025). Fluoroquinolone consumption and resistance after an Antibiotic Stewardship Team intervention—An interventional study in a single hospital in southern Poland from 2018–2023. Am. J. Infect. Control.

